# Rhinocerebral Mucormycosis in a Patient With Diabetes: A Rare but Critical Infection in the United Arab Emirates

**DOI:** 10.7759/cureus.80807

**Published:** 2025-03-19

**Authors:** Zeeshan Khan, Mohd Rafiw Ahmed Mahen, Faiza Akhlaque, Roberto Puxeddu, Aneela Darbar, Mohammed Abdulraheem, Simantini Jog

**Affiliations:** 1 Internal Medicine, Kings College Hospital, Dubai, ARE; 2 General Medicine, Kings College Hospital, Dubai, ARE; 3 Otolaryngology - Head and Neck Surgery, Kings College Hospital, Dubai, ARE; 4 Neurosurgery, Kings College Hospital, Dubai, ARE; 5 Critical Care Medicine, Kings College Hospital, Dubai, ARE; 6 Microbiology, Kings College Hospital, Dubai, ARE

**Keywords:** brain abscess excision, endonasal endoscopic surgery, mucormycosis, rhinocerebral mucormycosis, rhino-orbito-cerebral mucormycosis

## Abstract

A middle-aged man with no chronic medical conditions presented to the emergency department with flu-like symptoms, breathlessness, and vomiting. Arterial blood gas analysis revealed high blood glucose and an elevated anion-gap metabolic acidosis, suggestive of diabetic ketoacidosis, prompting the initiation of treatment. Neurological examination revealed multiple cranial nerve palsies with cranial mononeuritis multiplex, raising suspicion of rhino-orbito-cerebral mucormycosis. Liposomal amphotericin B was started empirically. Nasal endoscopy and biopsy, along with microbiological evidence, confirmed mucormycosis. Treatment required a multidisciplinary approach involving both medical and surgical specialties. In this case, we utilized treatments beyond current guidelines, including retrobulbar and intrathecal amphotericin in addition to intravenous dual antifungal therapy. Multiple surgical interventions were also performed. Over time, this approach led to clinical and biochemical improvement, allowing the patient to be discharged safely.

## Introduction

Rhino-orbito-cerebral mucormycosis (ROCM) is a relatively rare condition [1]. However, the risk factors contributing to its development, such as diabetes, are highly prevalent in the United Arab Emirates (UAE). According to the International Diabetes Federation, the prevalence of diabetes in the UAE was reported to be 16.3%, compared to 9.3% worldwide [2]. Additionally, a higher incidence of ROCM was observed in patients with prolonged corticosteroid use during the COVID-19 pandemic [3]. There is a critical need for heightened awareness and early diagnosis of mucormycosis, particularly ROCM. This case highlights the aggressive nature of the disease and emphasizes the necessity of a multidisciplinary approach to management, along with tailored guidelines and protocols specific to the region to improve patient outcomes.

## Case presentation

A male in his mid-30s presented to the emergency department with complaints of abdominal pain and multiple episodes of vomiting over 24 hours, with a background of ongoing flu symptoms for the preceding two weeks. He had no past medical history and was not on any regular medications. Upon arrival at the emergency room, he exhibited mild tachypnea and tachycardia without hemodynamic compromise. He also reported left-sided facial fullness and numbness, and a subtle left-sided ptosis was observed.

Blood tests revealed an elevated white cell count and CRP and a random blood sugar level of 525 mg/dL. Arterial blood gas analysis showed a pH of 6.9 with low bicarbonate (6.2), indicative of high anion gap (32.2) metabolic acidosis (Table 1). Consequently, a diagnosis of diabetic ketoacidosis was established, and treatment was initiated according to British Joint Society guidelines. The respiratory virus panel tested negative for SARS-CoV and influenza.

**Table 1:** Blood tests on admission ALT: alanine transaminase, AST: aspartate aminotransferase, BUN/CREAT: blood urea nitrogen creatinine ratio, HBA1c: hemoglobin a1c (three monthly blood sugar average), CRP: C-reactive protein, Ph: potential of hydrogen

Test	Result	Reference
White cell count	16.8*10^3/microL	3.4-10.8*10 ^3/microL
Hemoglobin	16.8 g/dL	11.1-15.9 g/dL
Platelet count	435*10^3/uL	150-379*10^3/microL
Albumin	3.8 g/dL	3.5-5.5 g/dL
ALT	46 10 IntlUnit/L	0-44 IntlUnit/L
AST	38 14 IntlUnit/L	0-40
BUN/CREAT	20 mg/dL	6-20 mg/dL
Calcium	7.8 mg/dL	8.7-10.2 mg/dL
Chloride	105 mmol/L	97-108 mmol/L
Creatinine	1.30 mg/dL	0.76-1.27 mg/dL
Glucose fasting	525 mg/dL	70-100 mg/dL
HBA1c	12.5%	<5.6%
Bicarbonate	6 mmol/L	18-29 mmol/L
Potassium	3.1 mmol/L	3.5-5.2 mmol/L
Urea blood	7.14 mmol/L	2.1-8.5 mmol/L
CRP	47.70 mg/L	0-4.90 mg/L
Ph	6.9	7.35-7.45

**Figure 1:**
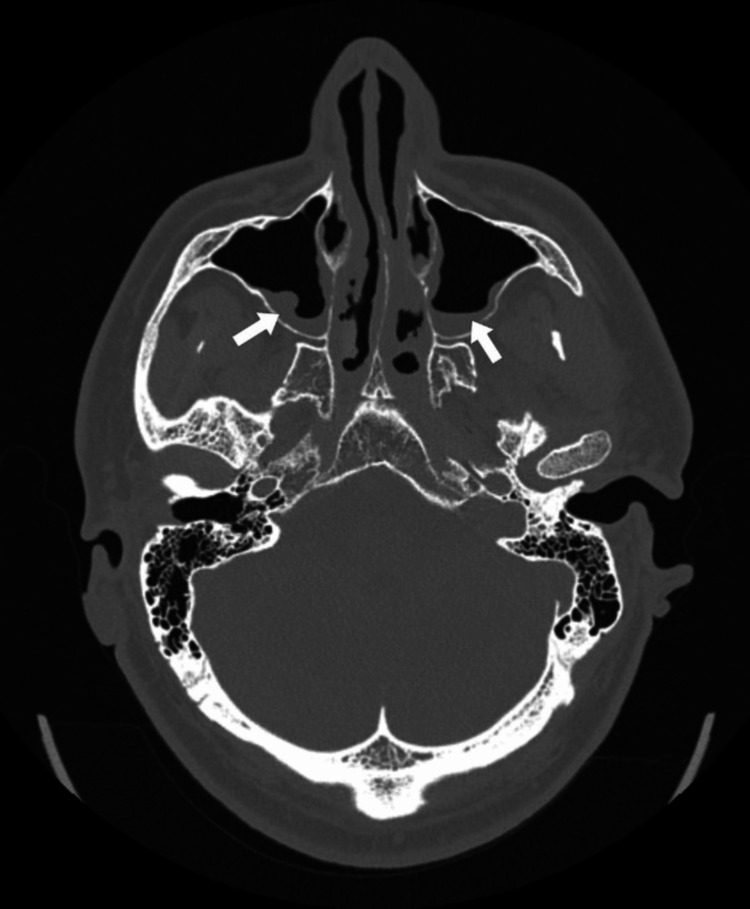
CT scan showing air fluid levels and mucosal thickening CT: computed tomography

**Figure 2:**
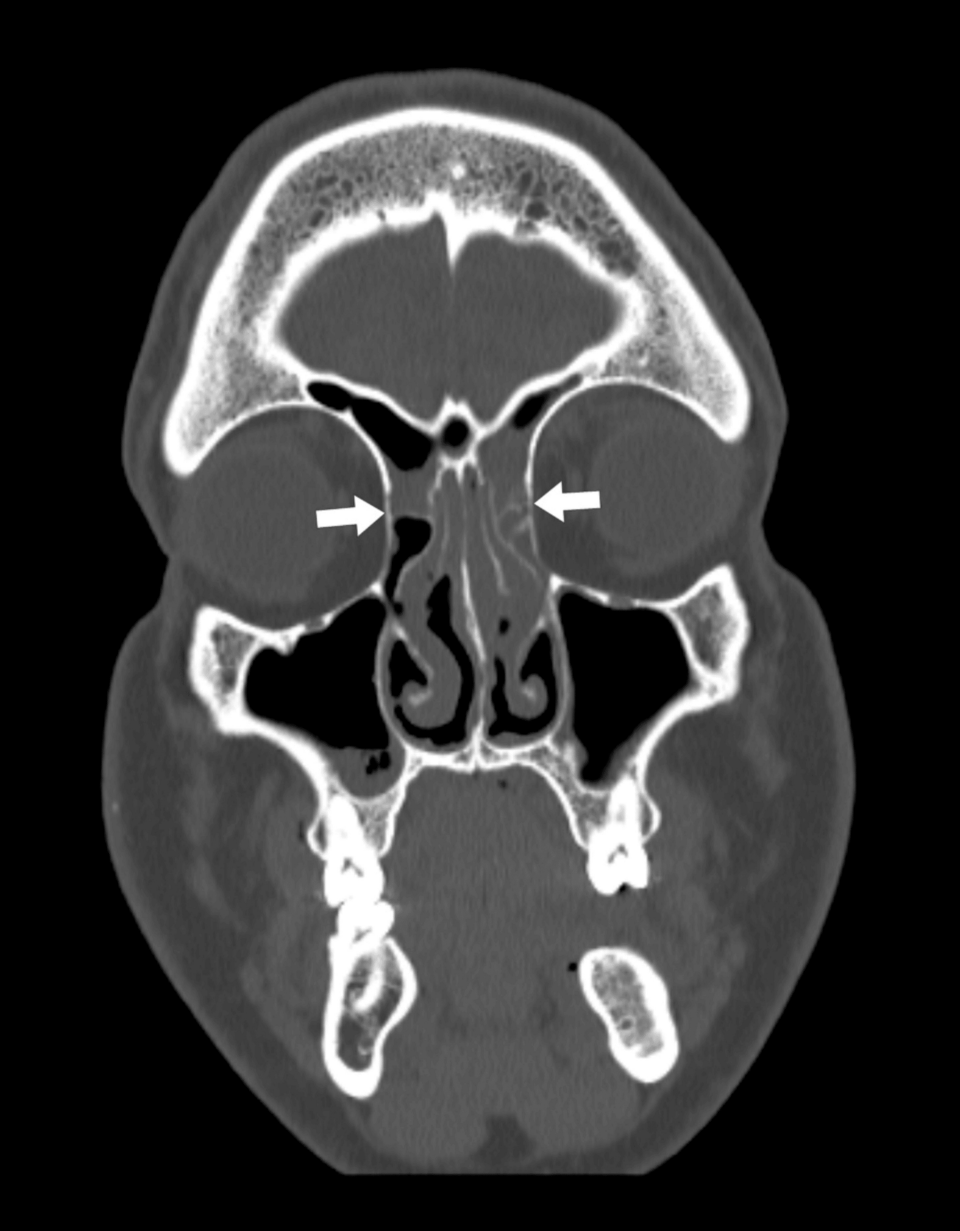
Bilateral opacification of ethmoidal cells

**Figure 3:**
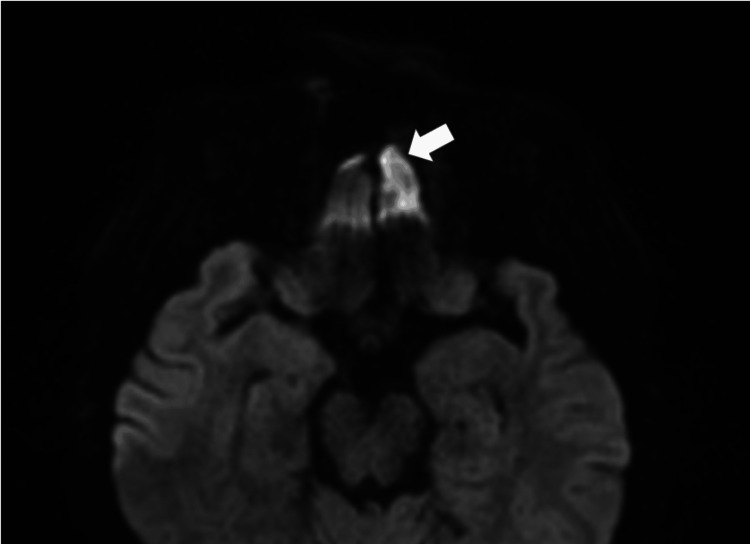
MRI diffusion restriction in the left gyrus rectus suggestive of inflammation/intracranial fungal invasion/or septic embolism MRI: magnetic resonance imaging

**Figure 4:**
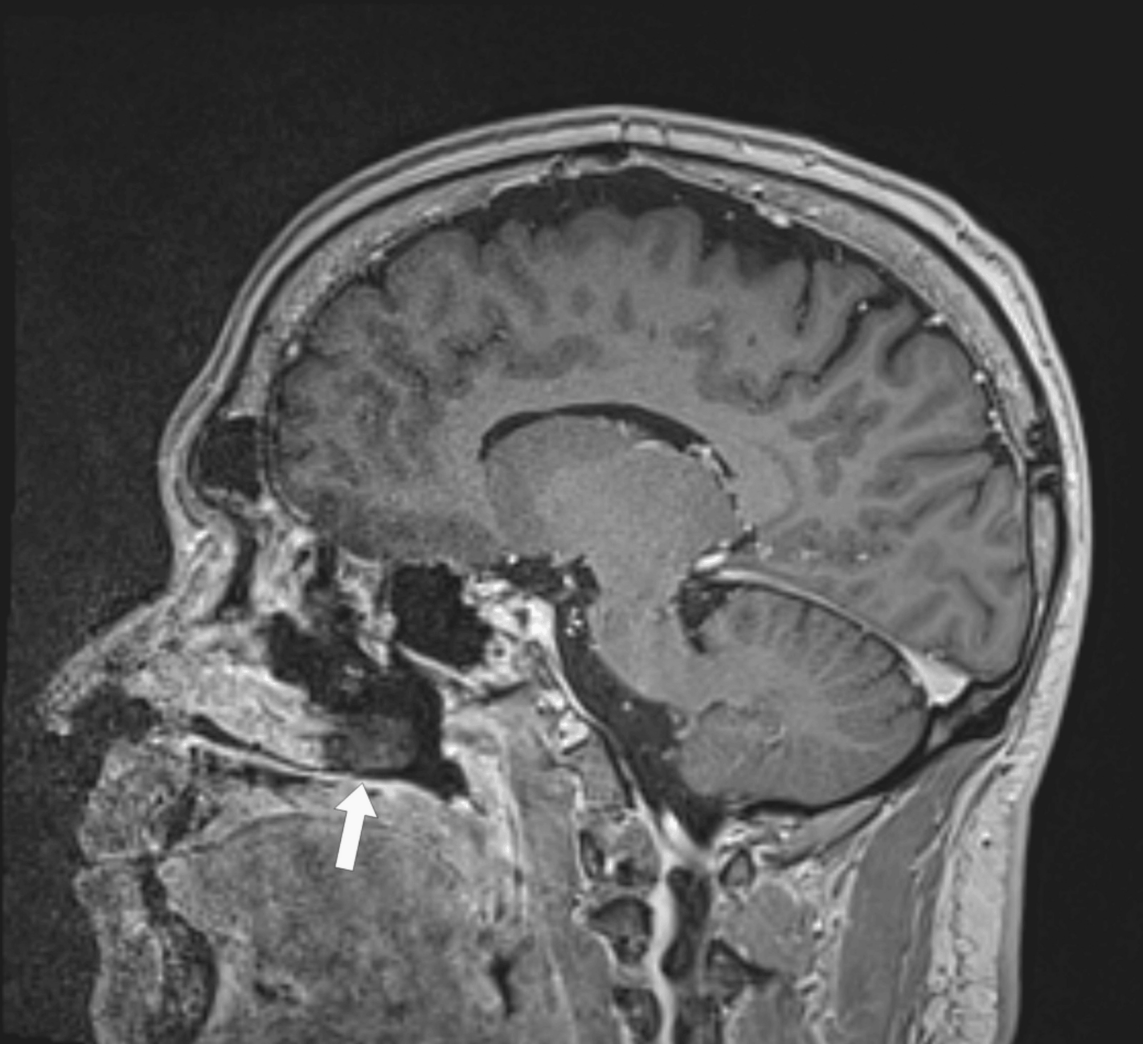
MRI showing hypointensity of the posterior aspect of the left inferior turbinate suggestive of fungal infection and invasion MRI: magnetic resonance imaging

A detailed neurological examination was performed, revealing left lateral rectus muscle paresis (sixth cranial nerve (CN)), bilateral superior recti muscle paresis (third CN), a right-sided facial droop with forehead involvement (seventh CN), and isolated numbness of the maxillary branch on the left side (fifth CN). Meningeal signs were negative. A suspicion of cranial mononeuritis multiplex due to leptomeningeal involvement was raised, with the presentation suggesting the possibility of mucormycosis. CSF analysis showed 49 cells with a 99% lymphocytic predominance. Liposomal amphotericin B (L-AMB) was empirically initiated at 5 mg/kg. CSF culture and beta-D-glucan testing yielded negative and low levels, respectively; Fungitell was 31.25 (<60, non-reactive). Given the complex neurological findings, a CT scan of the sinuses was initially conducted, followed by an MRI of the brain with contrast within 24-28 hours (Figures 1-4). The findings are described below.

While the patient’s condition improved from a diabetic ketoacidosis perspective and the ketoacidosis resolved, his facial numbness was noted to be worsening. Despite being on broad-spectrum antibiotics and amphotericin, he continued to have a fever.

**Figure 5:**
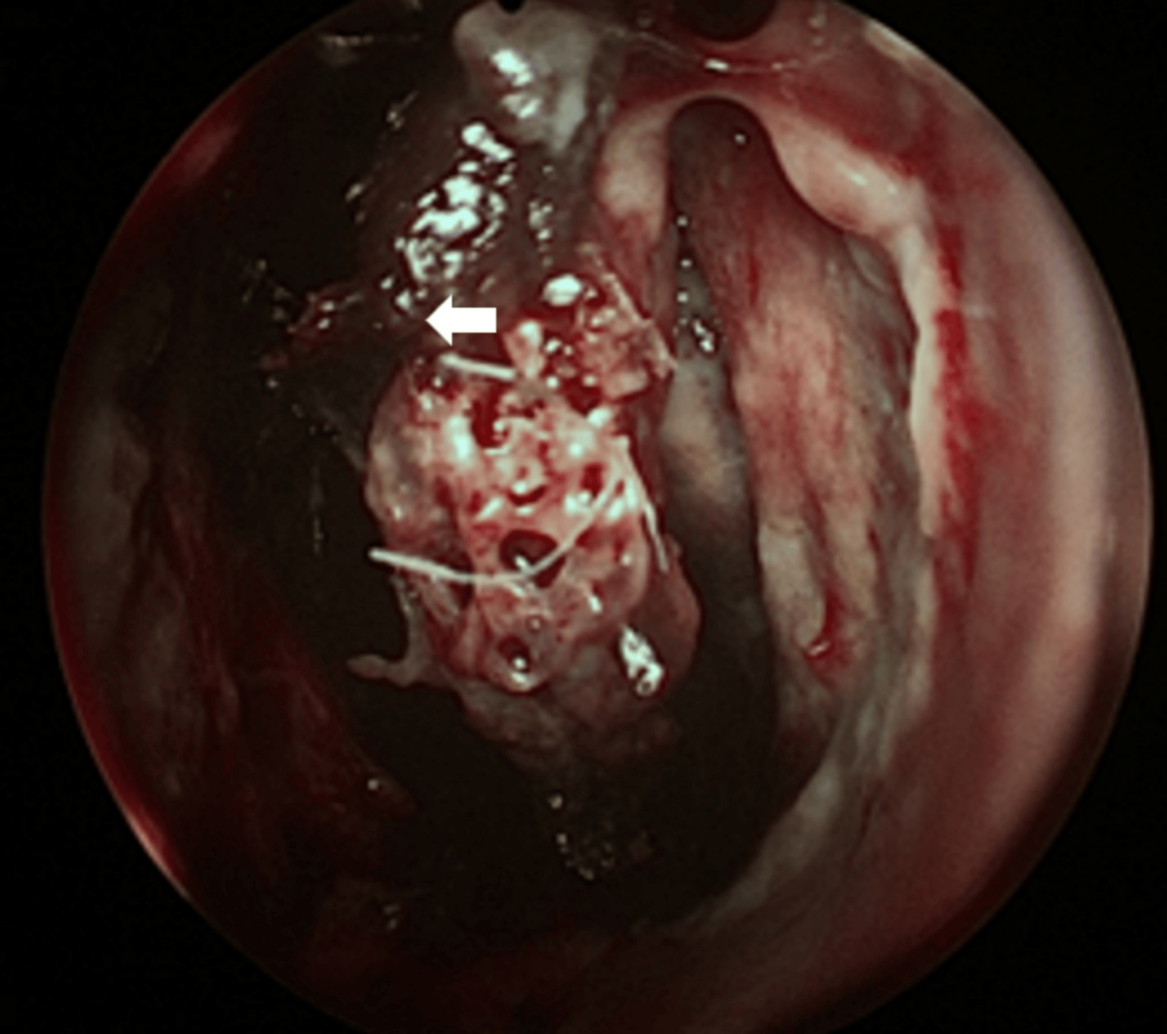
Black eschar left medial turbinate

**Figure 6:**
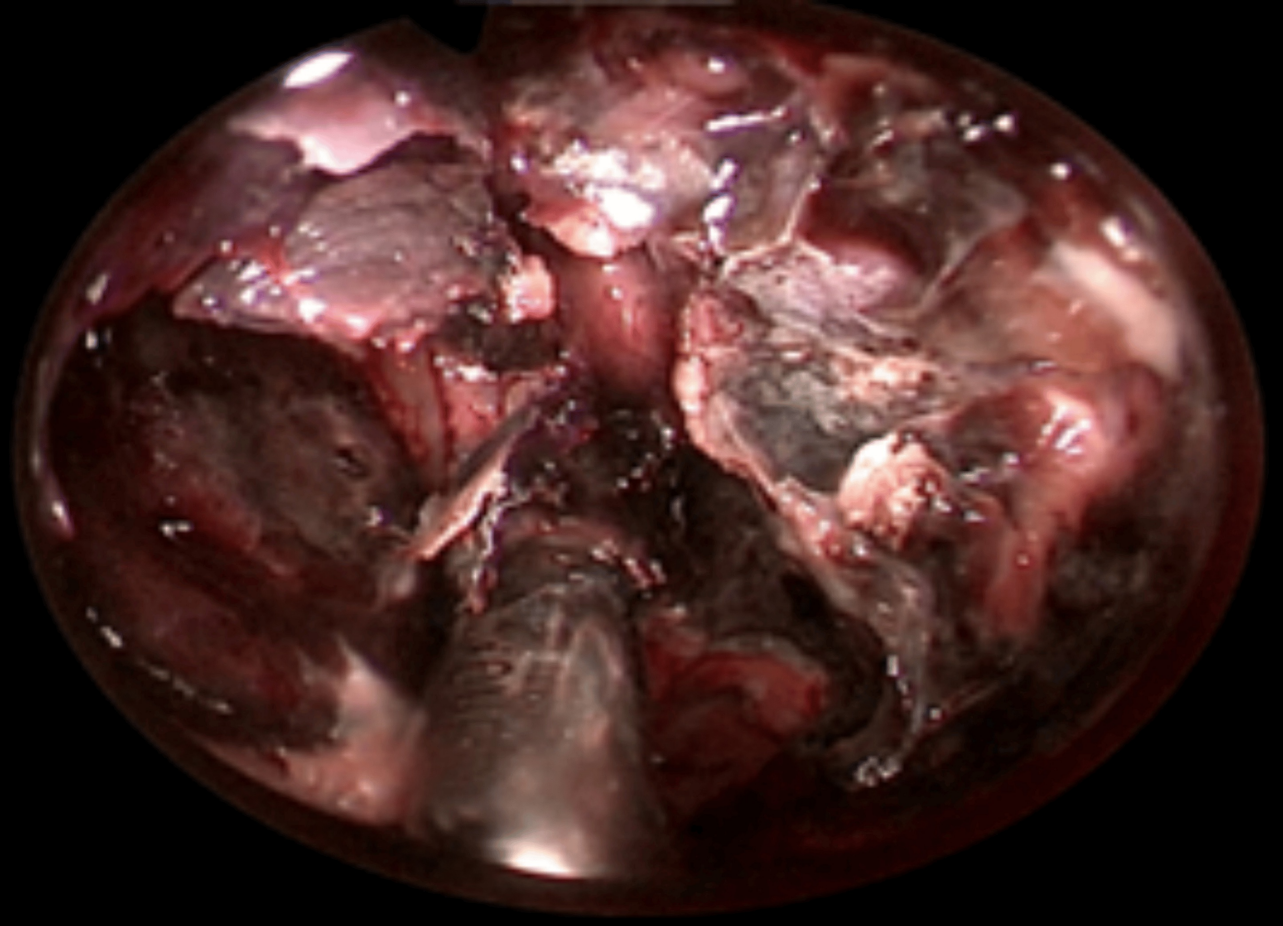
Endonasal extensive debridement with subtotal inferior turbinectomy, anteroposterior ethmoidectomy, sphenoidotomy, and subtotal resection of the nasal septum reaching the cribriform plate

Imaging was followed by nasal endoscopy, which revealed features of eschar, granulation, and purulent discharge predominantly in the left nasal cavity, findings highly suspicious for mucormycosis (Figures 5-6). Histopathological analysis of the nasal tissue showed fragments of inflamed hemorrhagic tissue with some outlines of seromucinous glands, within which branching septate fungal hyphae were observed. Given the findings suggestive of *Mucor*, L-AMB was increased to 10 mg/kg following the global guideline provided by the European Confederation of Medical Mycology-Mycoses Study Group Education and Research Consortium.

**Figure 7:**
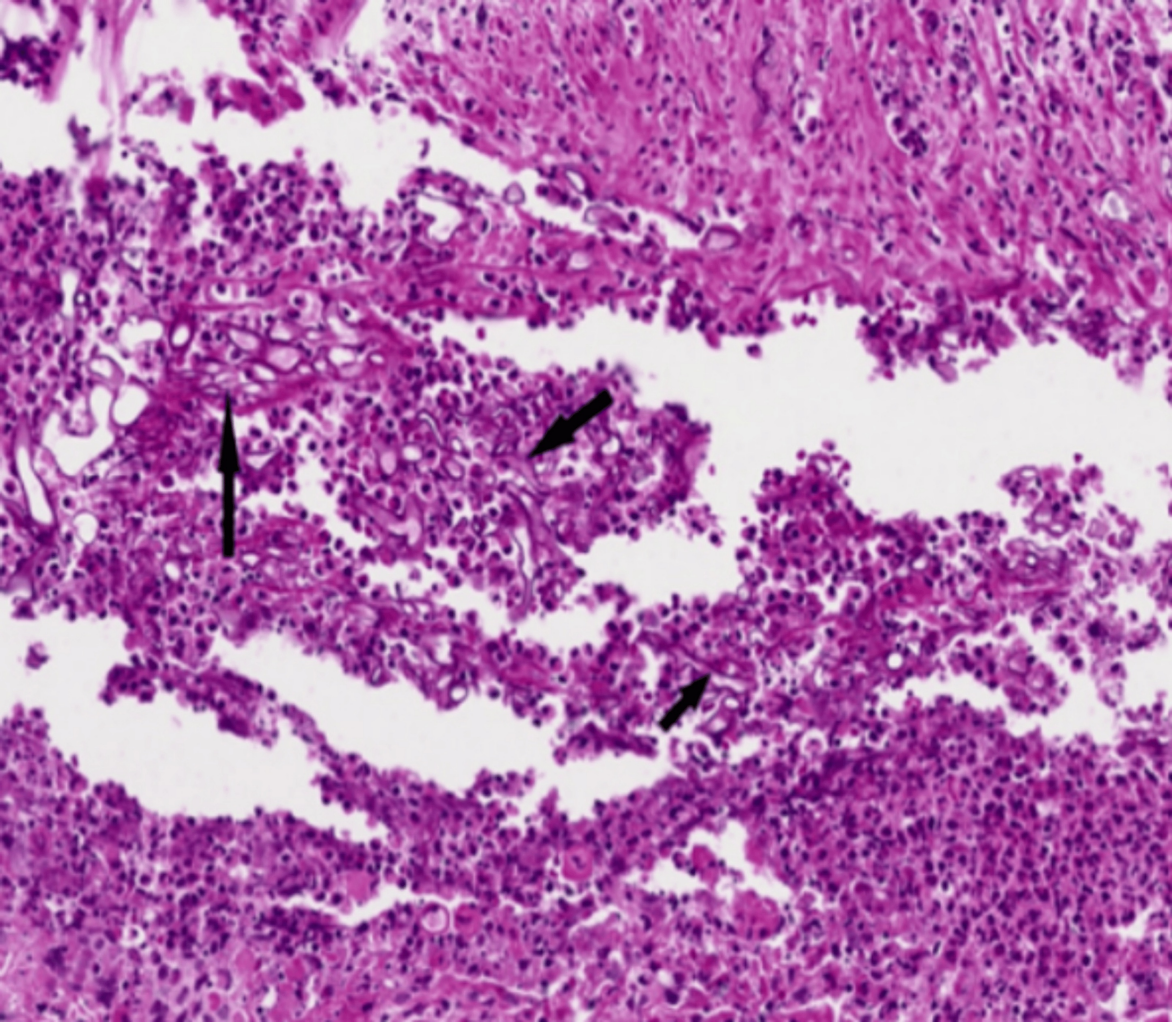
Inflammatory cell infiltrate with scattered fungal hyphae against a background of necrosis

After confirmation of mucormycosis, the patient underwent a staging CT scan, which revealed no further spread. This was followed by an emergency endoscopic endonasal extensive debridement of the necrotic mucosa and bone, including a subtotal inferior turbinectomy, anteroposterior ethmoidectomy, sphenoidotomy, and subtotal resection of the nasal septum up to the cribriform plate (Figure 7).

**Figure 8:**
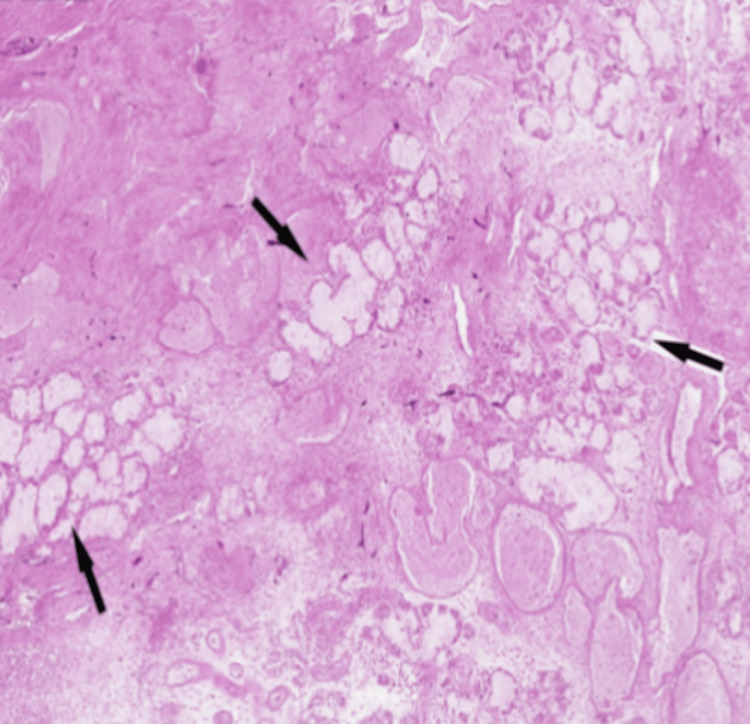
Extensive necrosis surrounded by inflammatory cells

**Figure 9:**
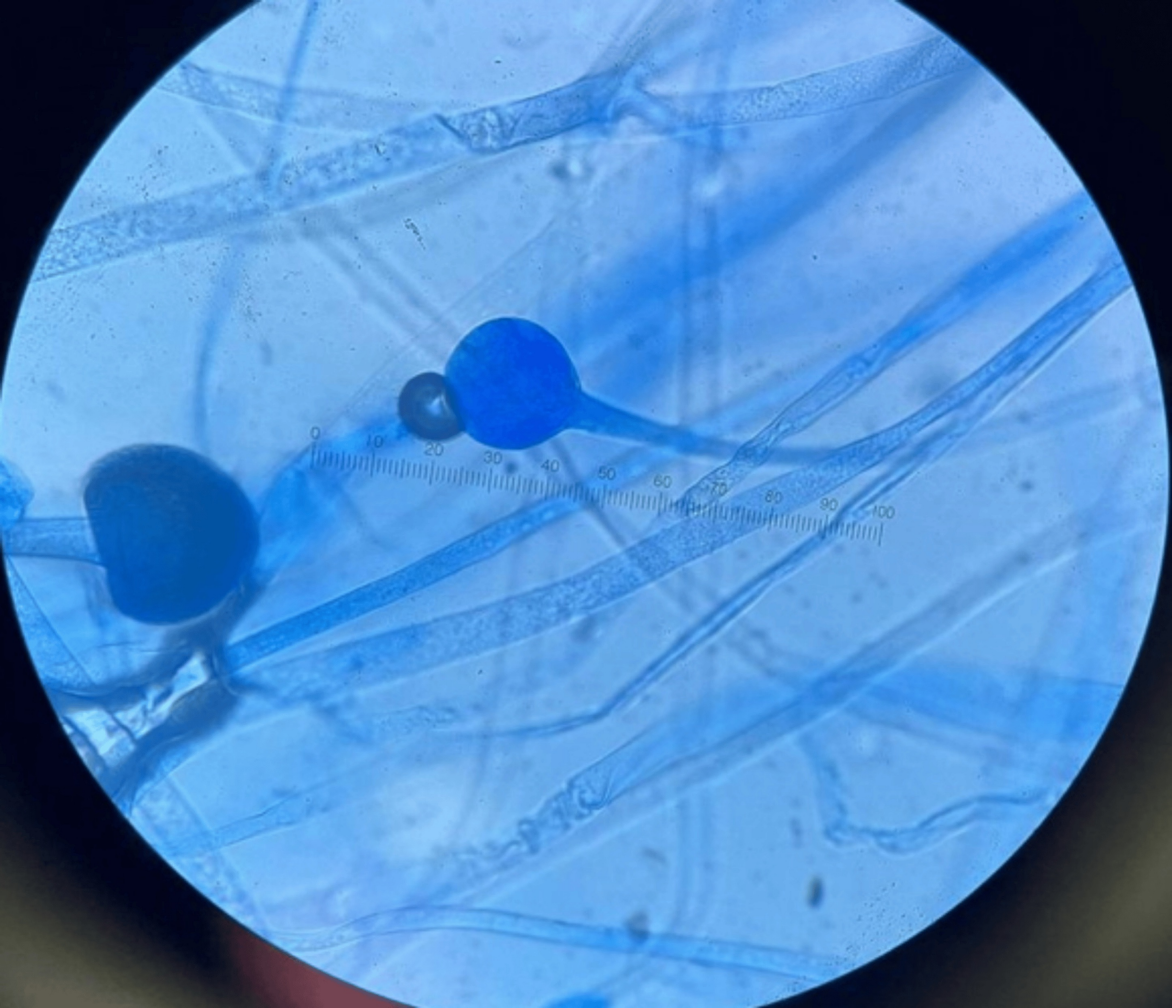
Fungal hyphae

The resected tissue was sent for histopathological analysis, which revealed fungal hyphae, spores, and florid acute inflammation with *Mucor* species. Figure 7 highlights the presence of hyphae (black arrows), Figure 8 shows extensive necrosis surrounded by inflammatory cells, and Figure 9 depicts fungal hyphae.

**Figure 10:**
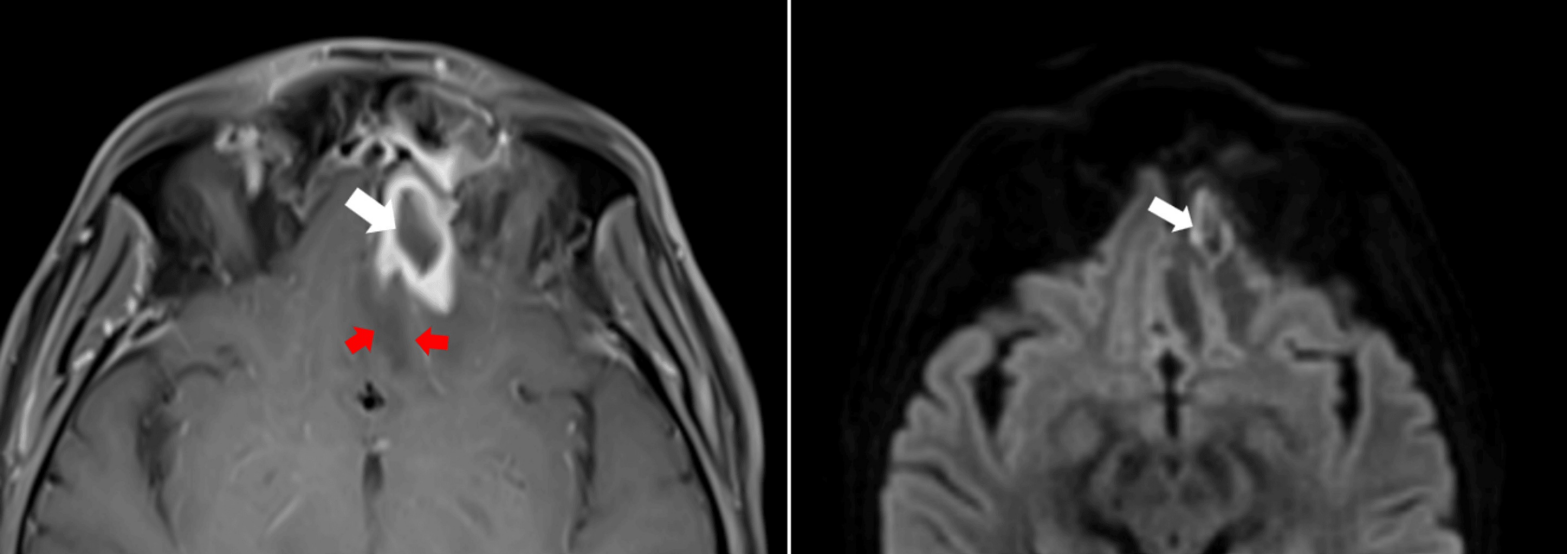
Peripheral enhancing lesion with perilesional edema without central diffusion restriction suggestive of fungal abscess

A subsequent MRI performed within five days of the initial imaging revealed a progressive lesion in the left base-frontal area, with a ring-enhancing lesion approximately 2 × 1.5 cm in size, indicative of a fungal abscess (Figure 10). A neurosurgical opinion was sought, and a left eyebrow craniotomy was performed, along with cranialization of the left frontal sinus, removal of the left frontal sinus mucosa, and debridement of the brain abscess.

Given the overall poor prognosis despite ongoing antifungal treatment and the need for repeated debridements, a multidisciplinary team meeting was held. This involved specialists from clinical microbiology, internal medicine, critical care, neurosurgery, otolaryngology, and neuroradiology to explore aggressive medical and surgical treatment options. These included nasal irrigation with amphotericin, intrathecal and retrobulbar amphotericin, dual intravenous antifungals, and exenteration.

Based on the recommendations, amphotericin was decreased to 5 mg/kg while intravenous isavuconazole (200 mg daily) was added. Alongside IV therapy, the patient received three doses of 3.5 mg retrobulbar L-AMB in the left eye and two doses in the right eye over one week. Due to the smaller-sized ventricles, we could not administer the intrathecal dose into the CSF via the ventricle using an external drain. Instead, we relied on 10 mg/day of intrathecal L-AMB via a lumbar drain over 14 days. Additionally, the nasal cavities and sinuses were irrigated with an amphotericin B solution using Merocel soaked in amphotericin B (100 mg in 250 ml of saline). The progression of treatment is outlined in Table 2.

**Table 2:** Progression and duration of treatment with antifungal medications L-AMB: liposomal amphotericin B, IV: intravenous

Medication sequence	Medication name	Duration of treatment
1	5 mg/kg IV L-amphotericin B	5 days
2	7.5 mg/kg IV L-amphotericin B	4 days
3	10 mg/kg IV L-amphotericin B	12 days
4	5 mg/kg IV L-amphotericin B + isavuconazole 200 mg + retrobulbar and intrathecal L-AMB injection	40 days IV along with three retrobulbar injections in the left eye and two in the right eye, plus 14 days of Intrathecal injections
5	Oral isavuconazole 200 mg	Stopped after 11 months

Over the next few weeks, he made a remarkable recovery, with improved headaches, decreased numbness, no neurological deficits, and stable lesions on MRI. He was eventually discharged with follow-up care involving weekly brain and sinus imaging, as well as blood tests, including CBC, CRP, and renal and liver function tests. Gradually, the follow-up interval was extended to monthly and then to every three months.

**Figure 11:**
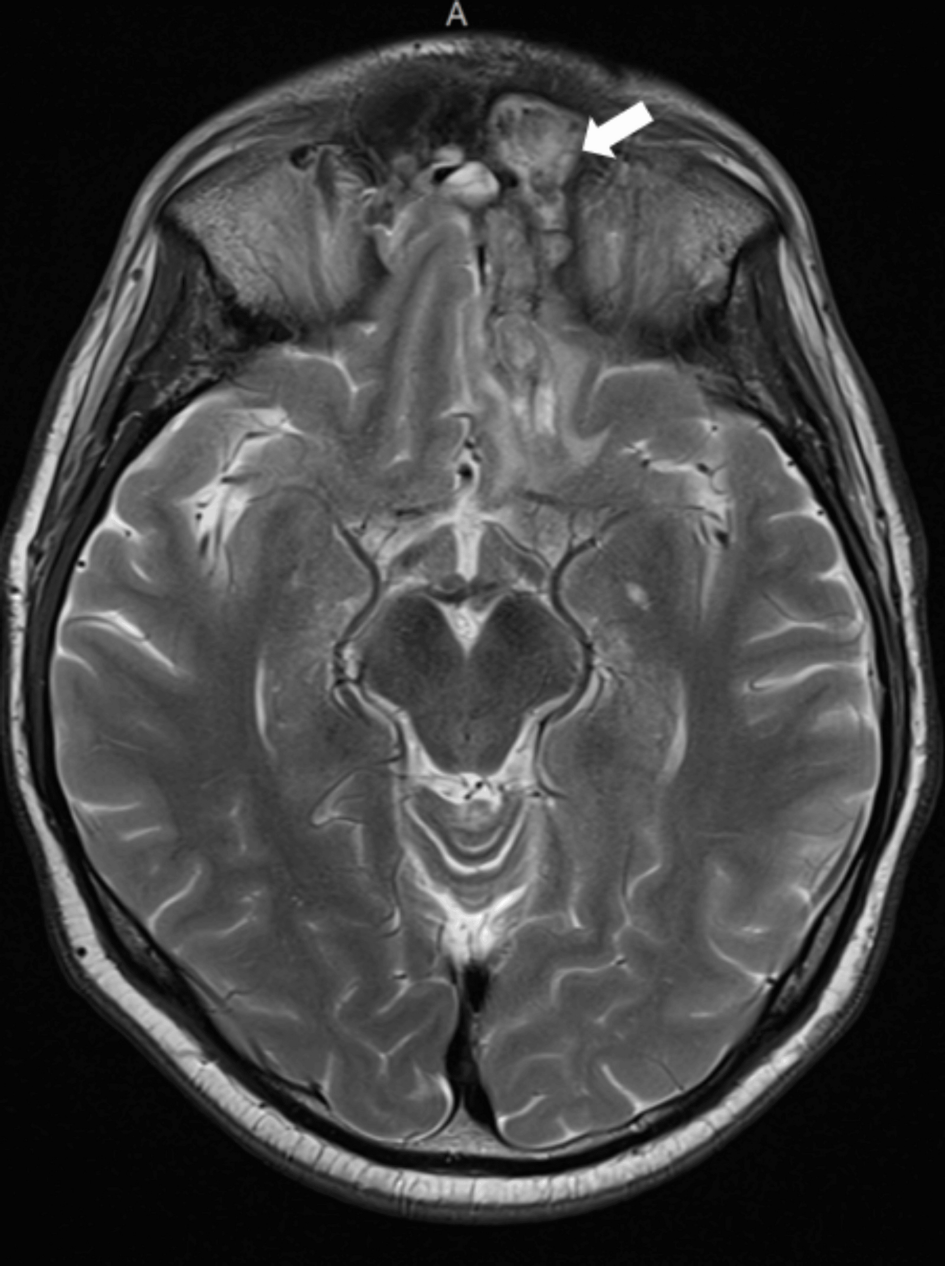
Three months post-discharge showing reduced perilesional edema and no signs of disease progression

**Figure 12:**
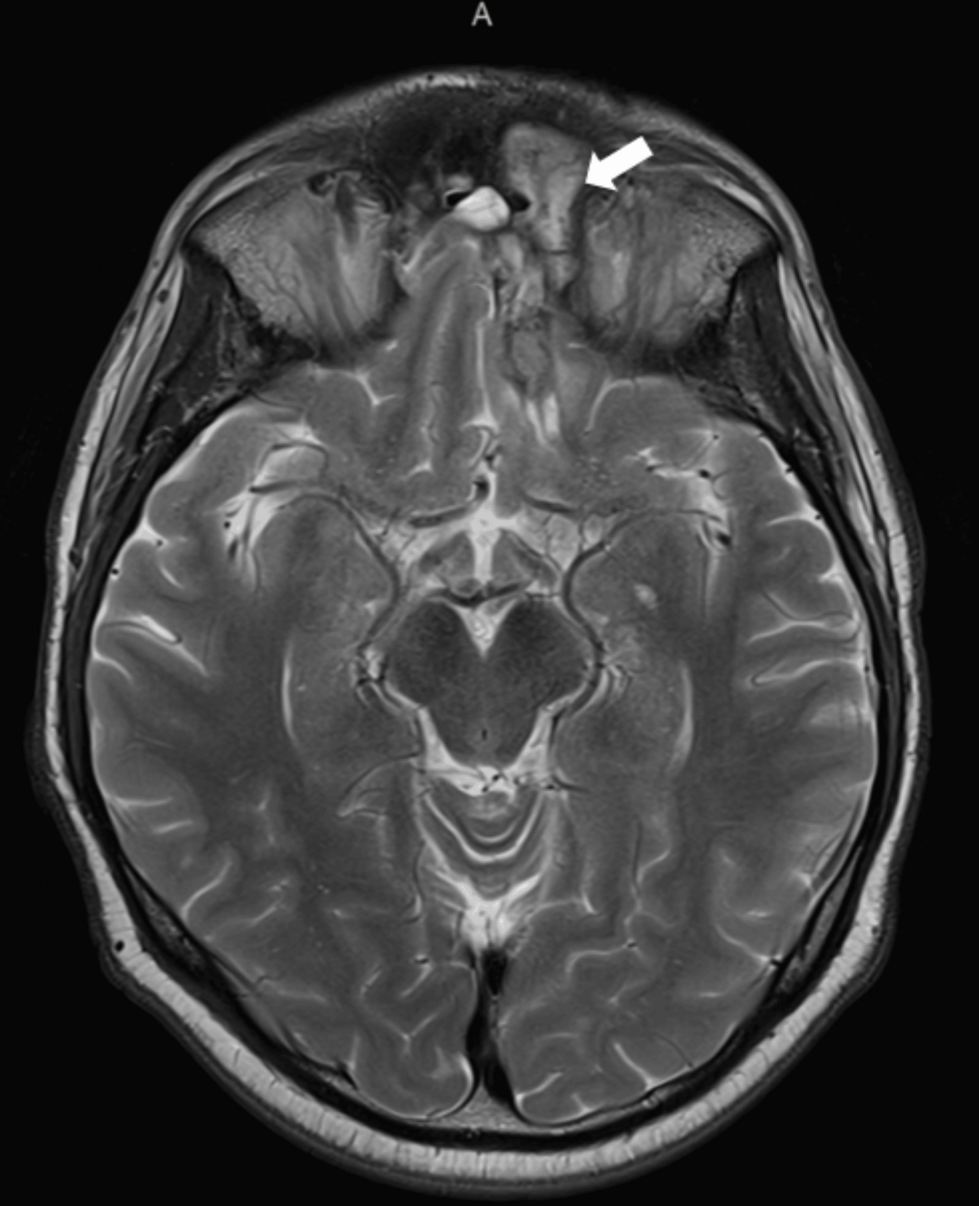
Six months post-discharge showing further decrease in size of surgical cavity and lack of disease progression

**Figure 13:**
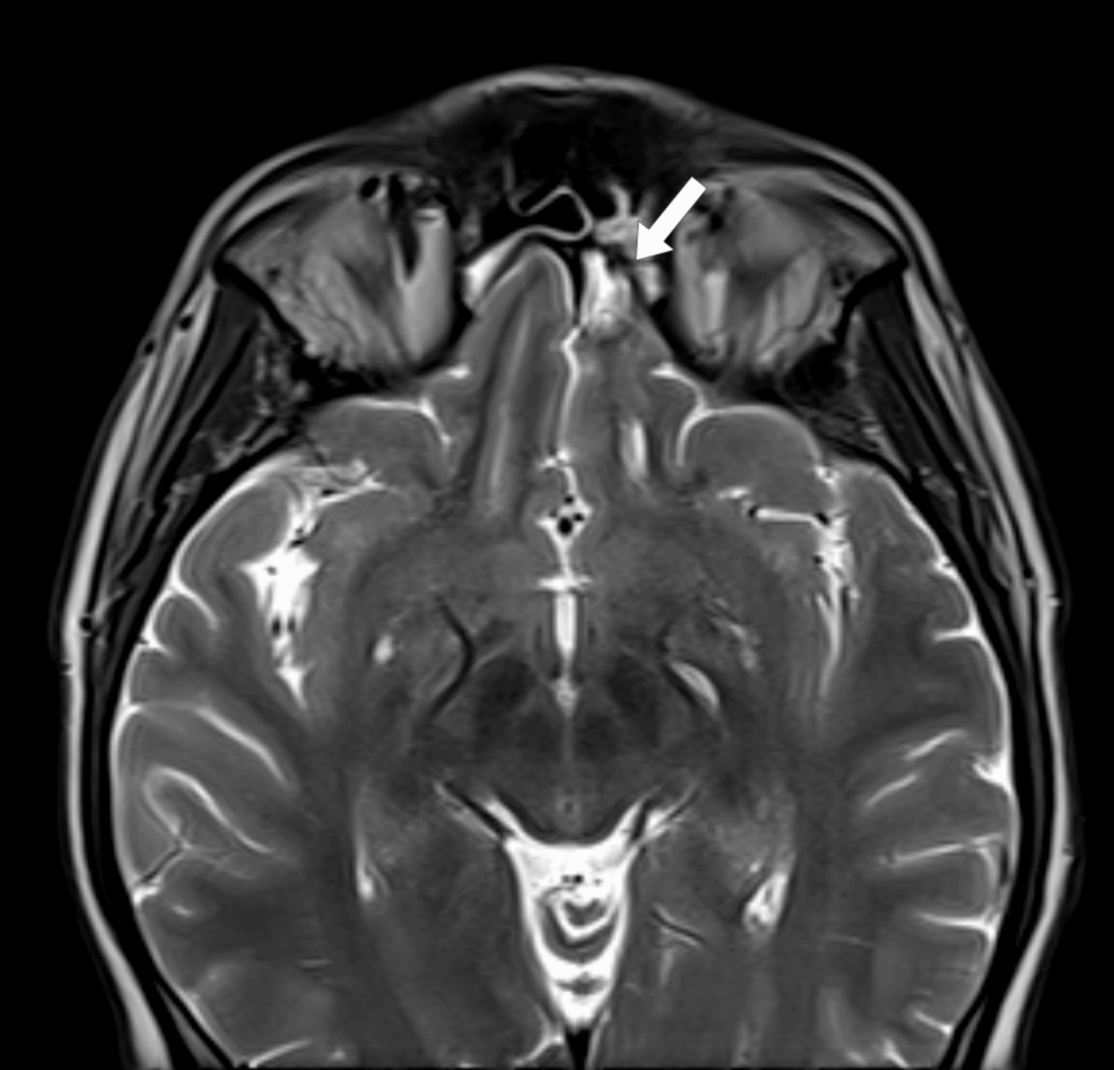
Nine months post-discharge showing mild area of postoperative changes with no evidence of fungal infection

MRI imaging at three, six, and nine months showed no worsening (Figures 11-13). By the ninth month, the area of diffusion restriction previously identified in the cortex of the gyri recti was no longer visible, and the involvement of the CNs on imaging appeared stable. A progressive decrease in the size of the surgical cavity and changes like sinus mucosal thickening were also noted.

**Figure 14:**
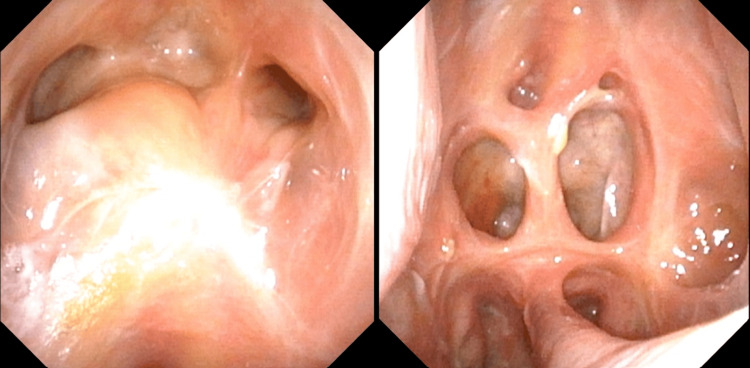
Postoperative endoscopic view after one year from surgery showing no recurrence

As he remained stable, oral isavuconazole 200 mg was discontinued 11 months post-discharge, with a mean duration of 330 days. His diabetes was well controlled, with no reported symptoms, normal inflammatory markers, and stable imaging on MRI. Additionally, the postoperative endoscopic view showed no recurrence (Figure 14).

## Discussion

ROCM is an aggressive infection caused by saprophytic fungi of the order *Mucorales*, characterized by broad hyphae, irregular branching, and rare septations [4] in the class *Zygomycetes*. The disease typically exhibits tropism toward the paranasal sinuses but can present with various manifestations through its spread to adjacent tissues [5]. ROCM involves infection of the paranasal sinuses, orbital tissue, including the optic nerve, and the brain. It is associated with diabetes mellitus, diabetic ketoacidosis, immunocompromised states, corticosteroid use, and COVID-19 [6]. Our patient initially presented with diabetic ketoacidosis secondary to undiagnosed type 2 diabetes mellitus.

Ketoacidosis is a well-known risk factor for mucormycosis, as it promotes conditions that facilitate fungal invasion and spread. In an acidic environment, iron is released from its binding proteins, such as transferrin, making it more accessible to pathogens like *Mucor*, which rely on iron for growth and survival. This creates an environment that significantly increases the likelihood of developing ROCM in individuals with ketoacidosis.

Mucormycosis typically begins when spores are inhaled into the paranasal sinuses. After inhalation, the fungus proliferates in the sinuses and can extend to the orbit through direct invasion or the nasolacrimal duct. The fungus spreads through the invasion of blood vessels, which is potentiated by damage to endothelial cells and extracellular matrix proteins lining the blood vessels. This process leads to thrombosis and necrosis [7]. From there, it can reach the brain through several pathways, including spread from the orbital apex, the cavernous sinus, the cribriform plate, or the bloodstream [8].

The precise incidence and prevalence of infections caused by these environmental pathogens remain unclear [9]. Although the incidence of mucormycosis in the UAE is not documented in available studies, data from the MENA region provide a useful context for comparison. A comprehensive review identified 310 cases in the region, rising from 23 cases before 1990 to 127 in the 2010s. Iran reported the highest number of cases (74), followed by Israel (63), Tunisia (49), Lebanon (28), Saudi Arabia (28), Egypt (20), Iraq (11), and Qatar (10), while other countries reported fewer than 10 cases each. In Oman, the annual incidence was estimated at 0.38-0.69 cases per million population before the COVID-19 pandemic [10,11].

Mortality rates can vary between 40% and 80%, depending on the specific clinical syndrome [12]. Hence, early diagnosis and initiation of treatment can improve prognosis. The European Confederation of Medical Mycology, in cooperation with the Mycoses Study Group Education and Research Consortium, has provided the latest recommendations for treating mucormycosis. The guidelines dictate using L-AMB at 5-10 mg/kg as first-line therapy. It is also recommended that doses not be increased gradually over several days; the maximum dose should be administered from the first day. Infections that have not spread to the central nervous system (CNS) can be treated with 5 mg/kg of L-AMB, whereas 10 mg/kg of L-AMB is recommended for infections involving the CNS.

Other antifungals recommended by the guidelines include isavuconazole and posaconazole, which are often used as salvage therapy. However, no definitive data guides the use of antifungal agents in combination therapy [13]. In our case, due to the aggressive nature of the infection and the difficulty in controlling its spread, we had to go beyond the guidelines, implementing combination antifungal therapy along with transcutaneous retrobulbar L-AMB injections and intrathecal L-AMB injections.

Several studies have demonstrated that transcutaneous retrobulbar amphotericin B (TRAMB) is effective and beneficial in managing and controlling ROCM. Yadav et al. (2022) [14], Ramamurthy et al. (2022) [15], and Shakrawal et al. (2022) [16] all conducted retrospective analyses of ROCM patients treated with TRAMB. Their findings suggested that TRAMB, when used alongside guideline-recommended therapies, effectively helps control fungal orbital involvement and reduces the need for orbital exenteration. The use of transcutaneous retrobulbar injections was not a common practice until the COVID-19 pandemic. During this period, there was a reported increase in the incidence of ROCM, with the infection spreading to the orbital region and the brain [14,15]. Several case reports have documented the use of retrobulbar antifungal injections in the treatment of orbital fungal infections [17-19]. Safi et al. (2020) reported the successful use of TRAMB in treating ROCM [20]. We observed similar encouraging results in our patients. With the use of TRAMB, disease progression was effectively controlled, and, most notably, the patient could avoid orbital exenteration.

While intrathecal amphotericin B is effective in some case reports [20-22], its use for ROCM is not well established. No large randomized controlled trials are evaluating its efficacy specifically for this condition. Guidelines do not routinely recommend intrathecal amphotericin B as a first-line treatment. In ROCM, intrathecal amphotericin B is used to achieve higher drug concentrations in the CSF, overcoming poor systemic penetration and the blood-brain barrier. This approach ensures effective antifungal activity at the site of infection while potentially reducing systemic toxicity. Its use in our patient helped control the aggressive nature of the infection and prevented its spread.

## Conclusions

The management of ROCM requires close collaboration among multiple specialties, including clinical microbiology, internal medicine, critical care, neurosurgery, otolaryngology, and neuroradiology, to ensure timely and comprehensive care. In the face of aggressive infections like ROCM, strictly adhering to guidelines may be insufficient. A proactive and innovative approach can be pivotal in controlling the disease and improving outcomes.

There is a pressing need for randomized clinical trials to evaluate the role of combination therapy, including intrathecal and retrobulbar injections, in ROCM. Revising current guidelines to incorporate these strategies could significantly enhance the management of this devastating condition and help avoid facially destructive surgeries and orbital exenteration.
